# Comprehension of action negation involves inhibitory simulation

**DOI:** 10.3389/fnhum.2013.00209

**Published:** 2013-05-30

**Authors:** Francesco Foroni, Gün R. Semin

**Affiliations:** ^1^Cognitive Neuroscience Sector, SISSA - TriesteTrieste, Italy; ^2^Faculty of Social and Behavioral Sciences, Royal Netherlands Academy of Arts and Sciences, Utrecht UniversityUtrecht, Netherlands; ^3^Psychology Department, Koç UniversityIstanbul, Turkey

**Keywords:** negation, simulation of language, grounded cognition

## Abstract

Previous research suggests that action language is comprehended by activating the motor system. We report a study, investigating a critical question in this research field: do negative sentences activate the motor system? Participants were exposed to sentences in the affirmation and negation forms while the zygomatic muscle activity on the left side of the face was continuously measured (Electromyography technique: EMG). Sentences were descriptions of emotional expressions that mapped either directly upon the *zygomatic* muscle (e.g., “I am smiling”) or did not (e.g., “I am frowning”). Reading sentences involving the negation of the activity of a specific muscle (zygomatic major—“I am not smiling”) is shown to lead to the inhibition of this muscle. Reading sentences involving the affirmative form instead (“I am smiling”) leads to the activation of zygomatic mucle. In contrast, sentences describing an activity that is irrelevant to the zygomatic muscle (e.g., “I am frowning” or “I am not frowning”) produce no muscle activity. These results extend the range of simulation models to negation and by implication to an abstract domain. We discuss how this research contributes to the grounding of abstract and concrete concepts.

## Introduction

An important issue in cognitive sciences is how concepts are represented. A substantial amount of the research has focused on the representation of actions in language (e.g., Pulvermüller, 1999; Buccino et al., 2004; Pulvermüller et al., 2005a,b; Hauk et al., 2008; Vigliocco et al., 2011). The evidence to date supports the argument that linguistic stimuli referring to *actions* automatically activate motor processes. The supportive evidence comes from behavioral (e.g., Zwaan and Taylor, 2006; Fischer and Zwaan, 2008), neurophysiological studies (e.g., Pulvermüller, 2004, 2005; Buccino et al., 2005; Pulvermüller et al., 2005a,b; Filimon et al., 2007—see Hauk et al., 2008, for a review), fine-grained movement-kinematic measures (Gentilucci and Gangitano, 1998; Glover and Dixon, 2002; Boulenger et al., 2006), and electromyographic analyses of facial muscles (e.g., Winkielman et al., 2008; Foroni and Semin, 2009, 2011).

Thus, evidence on the embodied grounding of meaning suggests that sensorimotor simulations of the content described by linguistic utterances are an essential component of language comprehension. Interestingly, movement disorders can affect language processing in a highly specific, action-related manner. Individuals with motor neuron disease (MND) are reported, for instance, to have subtle difficulties in action understanding (Bak and Hodges, 2004). Similarly, using a primed lexical decision task it was found that patients with Parkinson's Disease (PD) had delayed responding to verbs, but not to other verbal material (Boulenger et al., 2008). However, research investigating the representation of action language and its comprehension has mainly relied on single words (e.g., verbs of action like kick, lick, pick, etc.) or affirmative sentences of such actions (John kicks the ball, etc.; e.g., Pulvermüller, 2004; Pulvermüller et al., 2005a,b; Tettamanti et al., 2005; Aziz-Zadeh et al., 2006; Ruschemeyer et al., 2007; Boulenger et al., 2009; Raposo et al., 2009).

An important extension of this work is to understand how the comprehension of a negated action is represented. Negation is undoubtedly a cornerstone of human reasoning because it refers to an abstract aspect of reality, namely the absence of a concept (e.g., Horn, 2001; Hasson and Glucksberg, 2006), because its presence allows us to reason by contradiction and because it provides the means “for assigning truth value, for lying, for irony or for coping with false or contradictory statements” (Horn, 2001, p. XIII). Thus, understanding how we comprehend negation can also contribute toward a more general understanding of how people construct and evaluate alternatives (Hasson and Glucksberg, 2006). Negation is of particular interest also because it presents a challenge for models suggesting that the motor system drives action processing. Can the absence of an action be represented as a motor process? Moreover, the examination of negation catapults the research on the representation of actions into the study of the role that motor systems play in processing *abstract* concepts, a problematic domain for grounded theories (cf. Barsalou, 2008; but see e.g., Glenberg et al., 2008). Simulation theories of language postulate that language comprehension is mediated by sensorimotor simulations of the action represented in language (Barsalou, 1999; Glenberg and Kaschak, 2002; Glenberg and Gallese, 2012).

Negation of actions has received increasing attention (see e.g., Kaup et al., 2006, 2007; Tettamanti et al., 2008; Christensen, 2009; Tomasino et al., 2010; Liuzza et al., 2011; Kumar et al., 2013). Tettamanti et al. (2008) and Tomasino et al. (2010), using functional magnetic resonance imaging (fMRI), found a partial deactivation in action-related areas during comprehension of negative sentences suggesting context modulation of the motor simulation. Liuzza et al. (2011), using Transcranial Magnetic Stimulation (TMS), report evidence suggesting that motor simulation processes underlying the embodiment may involve even syntactic features of language such as negation. Because of technical constraints, some authors, however, doubt that neuroimaging (e.g., Tomasino et al., 2010) and TMS data (Liuzza et al., 2011) are able to determine whether reduced motor activity occurs after an initial phase of motor activation or negation simply leaves the motor structures less active (cf. Aravena et al., 2012). For these reasons, Aravena et al. (2012) implemented a fine-grained temporal analysis using “grip-force” measurement to investigate negation. These authors found that action words in negative sentences had no effect on force-grip. Although the results are fascinating, the data remain ambiguous and the actual cause of the observed motor-system activity (or decrease thereof) during action word processing remains elusive (Kemmerer and Gonzalez-Castillo, 2010) if one considers the results obtained with electromyography (EMG; e.g., Winkielman et al., 2008; Foroni and Semin, 2009). Taken together, the studies on the processing of sentence negation have produced conflicting results. One of the reasons for this is probably to be found in the differences in experimental design and procedures (cf. Tomasino et al., 2010). For instance, while Tomasino et al. (2010) implement imperatives, others have implemented more complex sentences (Liuzza et al., 2011; Aravena et al., 2012). These studies also differ in their focus on what comprehension constitutes (reading, listening) as well as they differ in the stimulus material. In particular, even though fMRI results furnish excellent information regarding the brain areas involved, their temporal resolution is poor. On the other hand, results obtained with TMS and grip-force analyses may at least address this issue partially.

The present study was conducted to examine whether negation is represented as a motor process and was designed to investigate the somatic correlates of negation (i.e., spontaneous muscle activity). We compare processing sentences involving negation of actions with their affirmative counterparts in order to uncover if any somatic activity is recruited when processing negation. We focused on a specific muscle (i.e., *zygomaticus major*: “smiling muscle”) of participants while they were reading sentences that refer to either the activation of the zygomatic (e.g., I am smiling) or to its negation (e.g., I am not smiling). As controls, we used sentences that are associated to a different facial muscle (e.g., I am frowning). We choose this particular focus because there is reliable evidence that the affirmative verbal representation of emotional expressions activates the corresponding facial muscles (e.g., Winkielman et al., 2008; Foroni and Semin, 2009). The rationale for using EMG as a technique is that it furnishes a fine-grained temporal resolution of motor activation relative to reading comprehension from the stimulus onset onward without the limitation of a time window of interest necessary for TMS research.

Two types of sentences were constructed, namely sentences referring to zygomatic activity and those that do not. If the simulation argument that relies on the activation of the motor system processing generalizes to negation, then one would expect affirmative sentences to induce zygomatic activation (e.g., I am smiling; Foroni and Semin, 2009) and that their sentential negation (e.g., I am not smiling) should inhibit it (cf. Tettamanti et al., 2008; Tomasino et al., 2010). Sentences that do not refer to zygomatic activity both in their affirmative or negative form (e.g., I am [not] frowning) would not be expected to show activation or inhibition. An alternative simulation hypothesis can be derived from the work by Kaup et al. (2006, 2007). Based on this work, one would predict that negation is initially simulated in its affirmative form, producing zygomatic activation as the affirmative form does, and only subsequently a simulation of the negation form is obtained. If however, the simulation argument of action processing does not generalize to the negation of action then no specific zygomatic muscle activity would be expected for the relevant sentences that are negated. This current measurement method will allow us to provide a precise timeline of the somatic correlates of the comprehension of negation and will allow us to investigate two hierarchical questions. First, in line with the embodied hypothesis of motor simulation the question is: does the comprehension of negation entail motor simulation? A positive answer to this question would maintain that negation, an abstract and uniquely human operation, also engages the motor system. In the case of an affirmative answer, then a second question would prompt: which kind of simulation does negation entail?

According to a recent simulation models understanding a sentence involving negation is the product of a comparison between a simulation of the affirmative form of the sentence and subsequently the simulation of the negated sentence (Kaup et al., 2007; see also Christensen, 2009). However, this hypothesis does not need to be the only one. By looking at muscle activity measured by surface electrodes (i.e., EMG) and at its time-course it will be possible to answer to both the questions raised above. This technique, in fact, provides high temporal resolution of the possible motor-simulation induced by language comprehension. So far little research has been conducted on this issue. While Foroni and Semin (2009) used verbs of action connected to facial expression (e.g., to smile), a recent EMG study (Stins and Beek, 2013) considered verbs symbolizing various actions performed by arm and leg effectors. The authors record EMG of two upper body muscles (deltoideus and biceps brachii) and two lower body muscles (tibialis anterior and vastus medialis). The results indicated a weak moderation of the EMG activity by the congruency between verb action (relative to arm vs. leg) and site of the EMG measurement (upper body vs. lower body muscles). The pattern of moderation reported seems to be at odds with the simulation hypothesis. However, it is important to note that the motor neurons engaged in upper and lower body part movements are far less differentiated and sensitive compared to those neurons involved in facial expressions (Tassinary et al., 2007) making more difficult to show strong systematic effects involving these muscles. Moreover, since the overall EMG results were very modest and most of the expected results were not found, the possible implications of this work should be considered with caution. Nevertheless, the results of a moderation of EMG activity reinforce the idea that EMG is a useful technique to study the online crosstalk between language comprehension and motor system.

## Materials and methods

### Participants and stimulus material

Thirty native Dutch speakers (12 females; 26 right-handed; mean-age = 22.2) participated in the experiment. Stimulus sentences (derived from Foroni and Semin, 2009) were verbal representations of emotional expressions that mapped either directly upon the *relevant* facial muscle (e.g., “I am smiling”-zygomaticus major muscle) or did not do so—*irrelevant* (e.g., “I am frowning”). When examining a specific muscle and the neuro-physiological correlates of language comprehension one encounters the problem of limited number of predicates that are similarly mapped onto the same muscle. However, this does not need to be a limit of the present research; in fact, other research has successfully investigated language comprehension with a similarly limited set of stimuli (e.g., Aziz-Zadeh et al., 2006; Foroni and Semin, 2009). In the present experiment *relevant predicates* were (original dutch predicate between brackets): to smile (glimlachen), to laugh (lachen), to grin (grinniken). *Irrelevant predicates* were: to frown (fronsen), to cry (huilen), to whine (janken). Each relevant or irrelevant predicate was presented in the affirmative and negative form using the first person singular conjugation. An example of affirmative sentence is: “I am smiling” (Ik glimlach); an example of negative sentence is: “I am not grinning” (Ik grinnik niet). Thus, there were three relevant-predicate sentences and three irrelevant-predicate sentences and each was presented in affirmative and negative form (12 sentences in total). The target sentences were intermixed with filler sentences that maintain the same structure as the target sentences and were also formulated in affirmative and negative form (12 fillers in total). The data relative to the filler sentences were not included in the analyses and, thus, not discussed in the present work.

### Procedure, apparatus, and data preparation

Participants were tested individually in a soundproofed experimental chamber. The experiment was presented as investigating the interference between reading and the performance at a simple spatial classification task and the mediating role of skin conductance. Participant's task was to classify images of arrows according to where the arrow was pointing (left or right) after reading short sentences while their skin conductance was *supposedly* measured.

Each trial consisted of a fixation point (500 ms), baseline interval (3000 ms), stimulus sentence (whole sentence was presented at once and remained on the screen for 4000 ms). At the end of the reading time and 500 ms interval the image of an arrow appeared in the center of the screen and stayed on the screen until the participant reported whether the arrow were pointing toward left or right. Each arrow-type (left-pointing and right-pointing) was presented in different visual forms (e.g., pointing toward top-right portion of the screen or bottom-right portion of the screen; with or without an oval circling the arrow) to create variation in the classification task. The sentence-arrow matching was randomly determined for each participant. After participants responded to the arrow the trial ended. After an inter-trial interval (3000 ms) the next trial started.

Participants completed eight practice trials with a set of affirmative and negative sentences different from the test sentences (e.g., “I am jumping,” “I am not hitting”). After the practice session participants received 5 blocks consisting of 24 trials each (12 test sentences and 12 fillers sentences). The five repetitions were performed to compensate the reduced number of stimuli and the high variability of physiological measurement (see Fridlund and Cacioppo, 1986). The order of presentation was randomized for each participant within each block. Zygomatic activity on the left side of the face was measured continuously (EMG using miniature Ag/AgCl electrodes and Coulbourn-Isolated-Bioamplifier: Coulbourn Inc., Whitehall, USA) at a sample rate of 1000 Hz. The digitized signal was bandpass filtered from 10 to 450 Hz and then full-wave rectified. Due to the nature of the research question and based on previous investigations (e.g., Foroni and Semin, 2009), we focus our analyses on the EMG response of the first 1000 ms after stimulus presentation. EMG responses were expressed in microvolts as change in activity from pre-stimulus level (baseline), a standard data aggregation procedure in physiological measurements (Fridlund and Cacioppo, 1986). *Baseline level* was considered the mean activity over a 500 ms period pre-stimulus presentation. As the baseline was supposed to reflect the muscle activity during resting/relaxing state, for each trial a 500 ms period of steady activity (i.e., without artifacts and/or extreme variations) was identified within the last second before stimulus presentation. Change in activity compared to baseline was averaged over intervals of 200 ms giving rise to 5 *periods* of 200 ms each during the time interval considered. Trials were excluded when artifacts were present or a steady baseline was absent (excluded trials: 5.8%).

### Design and statistical analysis

The design was a three within-subjects factorial: *Sentence relevance* (relevant vs. irrelevant) × *linguistic form* (affirmative vs. negative) × *period* (5 time intervals of 200 ms). Dependent variable was the mean activation level of the zygomatic major muscle (baseline-corrected) for each time period by sentence relevance and linguistic form.

Geisser–Greenhouse conservative *F*-tests were used to reduce likelihood of positively biased tests (see Kirk, 1968; Dimberg et al., 2002). A priori comparisons between means were evaluated by *t*-tests. Positive values of the muscle activation after baseline correction indicate the activation of the zygomaticus compared to pre-stimulus baseline, and negative values indicate inhibition compared to pre-stimulus baseline.

We first report the results of the omnibus analyses of variance. Then, we report separately the results for relevant and irrelevant sentences. For each type of sentence we report the a priori comparisons between the activation level and the zero-level to determine if there is a significant activation (or inhibition) for each time period. Additionally, within relevant and irrelevant sentences, we also report a priori comparisons between means for the affirmative and negative form (e.g., activation of “relevant, affirmative sentences” vs. activation of “relevant, negative sentences” in each time period after stimulus onset). Then we compared separately “relevant, affirmative sentences” and “relevant, negative sentences” against their correspondent irrelevant counterpart. Finally, we report the results of the classification task performed by the participants after being exposed to each stimulus.

## Results

**Figure 1:**
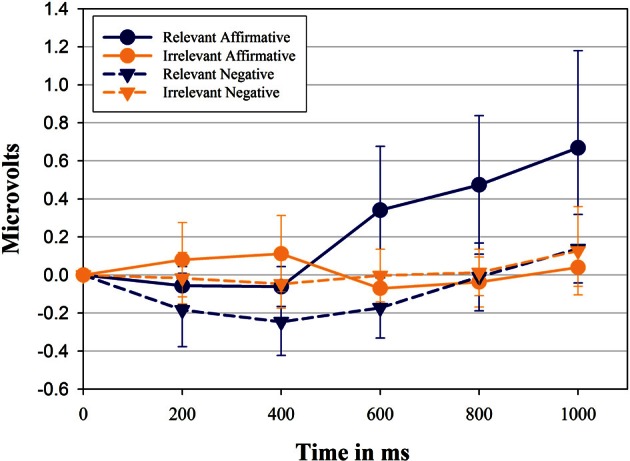
**Mean facial electromyographic (EMG) response and Confidence Intervals (CI 95%, as suggested by Cousineau, 2005) for the zygomaticus muscle.** Data represent the first 1000 ms of exposure to stimulus sentences and are plotted in intervals of 200 ms. Results are shown separately for each category of sentences and predicates used in the study. Positive values indicate the activation of the zygomaticus compared to pre-stimulus baseline, while negative values indicate inhibition compared to pre-stimulus baseline.

Figure 1 shows the change in zygomatic activity compared to pre-stimulus baseline as a function of sentence relevance, linguistic form, and period. The main hypothesis was supported by the significant 3-way interaction between *sentence relevance* × *linguistic form* × *period*, *F*_(2, 62)_ = 4.70, *p* = 0.011, η^2^_*p*_ = 0.14. Over time participants showed a differential activation of the zygomatic major muscle when presented with negative sentences compared with their affirmative counterparts, however, only when sentences are relevant to the muscle. Overall, zygomatic major activity increased over time, *F*_(2, 44)_ = 5.48, *p* = 0.013, η^2^_*p*_ = 0.16.

Affirmative sentences, in general, showed a larger activation compared to their negative counterparts, *F*_(1, 29)_ = 8.76, *p* = 0.006, η^2^_*p*_ = 0.23. As can also be seen from the *sentence relevance* × *period* interaction [*F*_(2, 63)_ = 5.09, *p* = 0.007, η^2^_*p*_ = 0.15] relevant sentences, in contrast to irrelevant sentences, induced a significant larger muscle activity over time. Finally, the interaction between *linguistic form* and *sentence relevance* was also significant [*F*_(1, 29)_ = 5.67, *p* = 0.024, η^2^_*p*_ = 0.16], indicating that in general affirmative sentences show a larger increase over time compare to negative sentences. Relevant and irrelevant sentences were then analyzed separately.

### Relevant sentences

Affirmative sentences show a significant activation of the zygomatic muscle (significantly higher than 0) in the last three time periods, (i.e., starting 400 ms after stimulus presentation, *p* = 0.046, 0.012, 0.012, respectively) while negative sentences show inhibition during the first 3 time periods (*p* = 0.06, 0.008, 0.032, respectively). Relevant sentences in affirmative form show a consistent and significantly larger activation of the zygomaticus muscle compared to their negative counterpart in each time period (*p* = 0.17, 0.011, 0.012, 0.005, 0.037).

### Irrelevant sentences

Irrelevant affirmative and irrelevant negative sentences produced no systematic zygomaticus muscle activity (all *t*-tests *ns*.) and they did not differ from each other at any point in time. We then compared relevant sentences against irrelevant sentences.

### Relevant sentences vs. irrelevant sentences

Relevant sentences in affirmative form show a significantly larger activation of the zygomatic muscle compared to the corresponding irrelevant sentences in the last three time periods (*p* = 0.022, 0.004, 0.009, respectively). Relevant sentences in negative form show a smaller activation of the zygomatic muscle compared to their irrelevant counterpart reaching significance in two of the first three time periods (*p* = 0.17, 0.06, 0.03, respectively).

### Classification task

To check the performance (RTs and accuracy) on the arrow-classification task reaction times and error percentage were analyzed separately in two 3-way analyses of variance with *sentence relevance* (relevant vs. irrelevant) × *linguistic form* (affirmative vs. negative) × *arrow direction* (left vs. right) as within subject factors. There was no significant effect of any one of the factors as main effect or in interaction on RTs or errors (all *p*s > 0.2).

## Conclusions

The findings reported here reveal that reading sentences negating actions is simulated as evidenced by the significant and extremely rapid *inhibition* of the relevant muscle (zygomatic). In contrast, affirmative sentences induce a significant activation of the same muscle. These findings advance the simulation argument underlying the action-related language processing view by generalizing it to negation.

As predicted, sentences irrelevant to the zygomatic (e.g., I am [not] frowning) did not induce any zygomatic activation or inhibition. These findings are in line with a neuromuscular mechanism for grounding negation. When considering only *affirmative sentences*, relevant sentences induced a significantly larger activation than irrelevant sentences. In sharp contrast, when considering only *negative sentences*, relevant sentences induced a significantly larger inhibition compared to irrelevant sentences. These results support the idea that the negation of an action verb is simulated by muscular inhibition. Negation, an abstract and uniquely human operation (Horn, 2001; Hasson and Glucksberg, 2006), also engages the motor system, however, by very rapidly inhibiting the relevant muscle action.

Two further elements of the stimuli and design add strength to this conclusion. First, the effects are not due to word order since negation is introduced after the action verb in Dutch (“*Ik lach**niet*”). Second, and more important, the observed inhibition effects were not due to a general inhibition induced by negation since the negated form of irrelevant sentences did not show any inhibition effects whatsoever. Thus, the physiological correlates of negation were dependent on the relevance of the sentence.

The present results are in line with studies using fMRI (e.g., Tettamanti et al., 2008; Tomasino et al., 2010). These investigations showed partial deactivation in action-related areas during comprehension of negative sentences suggesting context modulation of the motor simulation. In this vein, we show that comprehension of negation entails a fast inhibition of the relevant muscle. Recently, Kaup and colleagues advanced a theoretical model of the processing of negation (Kaup et al., 2007; see also Christensen, 2009), which assumes that the process of understanding a negative sentence (e.g., “John has not left”) can be traced back to a two step process of deviation-detection between two simulations (i.e., affirmative and negative form: “John has left” and “John has not left”) with the simulation of the negated sentence occurring around 1500 ms (or later) after the simulation of the affirmative one (occurring within the first 1500 ms). Our results do not support this model as negation shows a very quick inhibition of motor activity. Within this framework Liuzza et al. (2011), suggested that sentential negation could suppress the sensorimotor simulation of the (negated) action. Liuzza et al. implemented a TMS technique and reported lack of simulation contingent upon negation even in the time window (500–700 ms after stimulus presentation) where affirmative and negative sentences should not differ according to Kaup and colleagues. However, based on these results it is difficult to determine whether reduced motor activity occurs after an initial phase of motor activation or whether negation simply leaves the motor structures less active (cf. Aravena et al., 2012). According to our results, muscle inhibition occurs already around 500–700 ms after stimulus onset. Thus, our results suggest a neurophysiological model in which negation is encoded very quickly in terms of a reduced activation of the muscle whose activation is negated.

In the present research, we investigated sentences entailing the negation of action referring to emotional expressions. We were therefore able to examine directly the muscle involved in the expression (Tassinary et al., 2007). However, one may ask whether this pattern of muscle activation is specific to verbs mapping facial expressions because of their relation to emotional processing or whether these results could be generalized to any type of action verb (e.g., verbs involving arm movements). The reasons for raising this question are, first that there are inconsistencies in the literature on this issue and, second that in the domain of emotion contagion, muscle responses are reported also in the absence of visual processing (Tamietto et al., 2009) and seem to be independent from the specific body parts viewed. We think that verbs mapping facial expression may be simulated during language comprehension processes as other action verbs for several reasons.

First, the inconsistency in the literature seems largely due to differences in methodology. Secondly, the results reported by Tamietto and colleagues are not so easily compared to the present one. Tamietto et al. reported results from two patients showing muscle activation after visual stimuli presentation with a timeline consistent with emotional contagion (between 900 and 1200 ms). In sharp contrast, in the present experiment, the effects start already at 200 or 400 ms. Because of the difference in experimental population, task and set up one may wonder whether the results reported by Tamietto can be directly compared to the present ones. A third reason is the limited number of work implementing EMG technique in the investigation of the online crosstalk between language comprehension and motor system. The work providing clear-cut results in this domain almost exclusively relied on facial muscles and emotion-related stimulus material (Foroni and Semin, 2009; Niedenthal et al., 2009). The only exception has been the work by Stins and Beek (2013) but their work suggests caution. These authors considered verbs representing various actions performed by arm and leg effectors and reported moderation of the activity over upper body muscles (deltoideus and biceps brachii) and lower body muscles (tibialis anterior and vastus medialis) by the congruency between verb action (relative to arm vs. leg) and site of the EMG measurement (upper body vs. lower body muscles). While Niedenthal and colleagues and our works provide results supporting the simulation hypothesis, Stins and Beek do not find support for it. However, the results (and lack thereof) presented by Stins and Beek, are very weak and warrant some caution. Thus, the current state of the affairs do not allow a definitive conclusion in either direction. In order to support the notion that the comprehension verbs mapping facial expression are *not a special* case, a direct comparison between verbs referring to facial expressions and verbs referring to other actions should be a goal for future research.

Future research should investigate the differential somatic simulation of other linguistic features such as actor of the action (I am smiling vs. you are smiling vs. my friend is smiling). A recent investigation implementing TMS reports increased motor-evoked potentials for first person action-verb sentence and not for third person action-verb sentences suggesting specificity of motor involvement in language processing or at least contextual modulation (Papeo et al., 2011). Furthermore, simulation models of language comprehension could be also investigated in children in order to test the development of motor simulations during language processing. Finally, it would be important for future research to extend the range of simulation models also to other types of negations sentences (e.g., “the stapler is not on the table”) and further to other examples of abstract concepts such as “to ignore,” “to dream,” or “to hope.”

When examining a specific muscle and the neuro-physiological correlates of language comprehension often the number of suitable stimuli is limited. In this research we used six different predicates that were relevant or irrelevant to the zygomatic muscle. The limited number of stimuli used here is similar to the one selected in other research that successfully investigated language comprehension (e.g., Aziz-Zadeh et al., 2006; Foroni and Semin, 2009). Future research, however, should replicate these results with another (possibly larger) set of predicates to increase generalizability by implementing eventually the EMG measurement of other muscles (see Stins and Beek, 2013).

In the present research muscle reactions associated with affirmative and negative sentences showed different timelines and this result deserves further investigation particularly because it is at variance with behavioral evidence suggesting that the processing of affirmative sentences is faster than the one of negation sentences (Hasegawa et al., 2002). The data reported here show faster inhibitory activities (within 200 ms) compared to the activation response (starting at 400 ms). Considering the results from electrophysiological studies on semantic processing (e.g., Pulvermüller et al., 2005a,b; Hauk et al., 2006; Penolazzi et al., 2007), this fast inhibitory muscle response to the reading of negation sentences relevant to the muscle seem to suggest that negation is processed in early (within 200 ms) lexical-semantic stage compared to a late (within 400 ms) lexical-semantic stage. It should be noted that the sentences used in the present research are relatively short (2 or 3 words) allowing for fast reading time. The present results are not at variance with the suggestion that motor simulation precedes semantic decoding also supported by the temporal difference between automatic EEG response to semantic anomaly (i.e, N400) and the motor response (Friederici, 2002; Christensen and Wallentin, 2011). However, the reasons for such difference might reside in the neuro-anatomical differences of the processing of affirmation and negation (Carpenter et al., 1999; Hasegawa et al., 2002) or in the salience of the negative sentence in comparison to the “default mode” constituted by the affirmative sentences (Christensen, 2009).

Even though the present results do not directly speak to the causal role of sensory and motor activation/simulations in conceptual processing (see e.g., Mahon and Caramazza, 2008), they constitute an important step in inviting the examination of the neurophysiological and somatic underpinnings of the negation of action-related language and may serve in guiding future research on concrete and abstract concepts. These results also represent an important step forward in understanding how abstract concepts as well as concrete ones can be accommodated within embodied theories (cf. Barsalou, 1999; Boroditsky and Prinz, 2008; see also e.g., Glenberg et al., 2008; Kousta et al., 2011; Kiefer and Pulvermüller, 2012).

Oftentimes there is a separate treatment of concrete and abstract concepts in the literature. On the one hand, concrete categories such as actions are deemed to be best dealt with simulation models (e.g., Barsalou, 1999, 2008; Fischer and Zwaan, 2008; Glenberg and Gallese, 2012). On the other hand, research with abstract categories mainly resorts to Conceptual Metaphor Theory (CMT, Lakoff and Johnson, 1980, 1999) or related models (e.g., Boroditsky, 2000; Boroditsky and Prinz, 2008). Negation as we have examined here does not fall into the same type of abstract categories addressed by CMT. Nevertheless, the evidence we advanced here suggests that an abstract concept involving the absence of an action is also clearly embodied in terms of engaging an inhibition of the motor system very much as proposed by simulation models of embodiment.

### Conflict of interest statement

The authors declare that the research was conducted in the absence of any commercial or financial relationships that could be construed as a potential conflict of interest.

## References

[B1] Aravena P. Delevoye-Turrell Y. Deprez V. Paulignan Y. Frak V. Nazir T. (2012). Grip force reveals the context sensitivity of language-induced motor activity during “action words” processing: evidence from sentential negation. PLoS ONE 7:e50287 10.1371/journal.pone.0050287 23227164 PMC3515598

[B2] Aziz-Zadeh L. Wilson S. M. Rizzolatti G. Iacoboni M. (2006). Congruent embodied representations for visually presented actions and linguistic phrases describing actions. Curr. Biol. 16, 1818–1823 10.1016/j.cub.2006.07.060 16979559

[B3] Bak T. H. Hodges J. R. (2004). The effects of motor neurone disease on language: further evidence. Brain Lang. 89, 354–361 10.1016/S0093-934X(03)00357-2 15068918

[B4] Barsalou L. W. (1999). Perceptual symbol systems. Behav. Brain Sci. 22, 577–609 11301525 10.1017/s0140525x99002149

[B5] Barsalou L. W. (2008). Grounded cognition. Annu. Rev. Psychol. 59, 617–645 10.1146/annurev.psych.59.103006.093639 17705682

[B6] Boroditsky L. (2000). Metaphoric structuring: understanding time through spatial metaphors. Cognition 75, 1–28 10.1016/S0010-0277(99)00073-6 10815775

[B7] Boroditsky L. Prinz J. (2008). What thoughts are made of, in Embodied Grounding: Social, Cognitive, Affective, and Neuroscientific Approaches, eds Semin G. Smith E. (New York, NY: Cambridge University Press), 98–116

[B8] Boulenger V. Hauk O. Pulvermuller F. (2009). Grasping ideas with the motor system: semantic somatotopy in idiom comprehension. Cereb. Cortex 19, 1905–1914 10.1093/cercor/bhn217 19068489 PMC2705699

[B9] Boulenger V. Mechtouff L. Thobois S. Broussolle E. Jeannerod M. Nazir T. A. (2008). Word processing in Parkinson's disease is impaired for action verbs but not for concrete nouns. Neuropsychologia 46, 743–756 10.1016/j.neuropsychologia.2007.10.007 18037143

[B10] Boulenger V. Roy A. C. Paulignan Y. Déprez V. Jeannerod M. Nazir T. A. (2006). Cross-talk between language processes and overt motor behavior in the first 200 ms of processing. J. Cogn. Neurosci. 18, 1607–1615 10.1162/jocn.2006.18.10.1607 17014366

[B11] Buccino G. Binkofski F. Riggio L. (2004). The mirror neuron system and action recognition. Brain Lang. 89, 370–376 10.1016/S0093-934X(03)00356-0 15068920

[B12] Buccino G. Riggio L. Melli G. Binkofski F. Gallese V. Rizzolatti G. (2005). Listening to action-related sentences modulates the activity of the motor system: a combined TMS and behavioral study. Cogn. Brain Res. 24, 355–363 10.1016/j.cogbrainres.2005.02.020 16099349

[B13] Carpenter P. A. Just M. A. Keller T. A. Eddy W. F. Thulborn K. R. (1999). Time course of fMRI-activation in language and spatial networks during sentence comprehension. Neuroimage 10, 216–224 10.1006/nimg.1999.0465 10417254

[B14] Christensen K. R. (2009). Negative and affirmative sentences increase activation in different areas in the brain. J. Neurolinguist. 22, 1–17

[B15] Christensen K. R. Wallentin M. (2011). The locative alternation: distinguishing linguistic processing cost from error signals in Broca's region. Neuroimage 56, 1622–1631 10.1016/j.neuroimage.2011.02.081 21385619

[B16] Cousineau D. (2005). Confidence intervals in within-subject designs: a simpler solution to Loftus and Masson's method. Tutorials Quant. Methods Psychol. 1, 42–45 10.3758/s13423-012-0230-1 22441956 PMC3348489

[B17] Dimberg U. Thunberg M. Grunedal S. (2002). Facial reactions to emotional stimuli: automatically controlled emotional responses. Cogn. Emot. 16, 449–472 10.1016/j.neuroimage.2004.10.013 15652310

[B18] Filimon F. Nelson J. D. Hagler D. J. Sereno M. I. (2007). Human cortical representations for reaching: mirror neurons for execution, observation, and imagery. Neuroimage 37, 1315–1328 10.1016/j.neuroimage.2007.06.008 17689268 PMC2045689

[B19] Fischer M. H. Zwaan R. A. (2008). Embodied language: a review of the role of the motor system in language comprehension. Q. J. Exp. Psychol. 61, 825–850 10.1080/17470210701623605 18470815

[B20] Foroni F. Semin G. R. (2009). Language that puts you in touch with your bodily feelings. The multimodal responsiveness of affective expressions. Psychol. Sci. 20, 974–980 10.1111/j.1467-9280.2009.02400.x 19594858

[B21] Foroni F. Semin G. R. (2011). When does mimicry affect evaluative judgment? Emotion 11, 687–690 10.1037/a0023163 21668115

[B22] Fridlund A. J. Cacioppo J. T. (1986). Guidelines for human electromyographic research. Psychophysiology 23, 567–589 3809364 10.1111/j.1469-8986.1986.tb00676.x

[B23] Friederici A. D. (2002). Towards a neural basis of auditory sentence processing. Trends Cogn. Sci. 6, 78–84 10.1016/S1364-6613(00)01839-8 15866191

[B24] Gentilucci M. Gangitano M. (1998). Influence of automatic word reading on motor control. Eur. J. Neurosci. 10, 752–756 10.1046/j.1460-9568.1998.00060.x 9749737

[B25] Glenberg A. M. Gallese V. (2012). Action-based language: a theory of language acquisition, comprehension, and production. Cortex 48, 905–922 10.1016/j.cortex.2011.04.010 21601842

[B25a] Glenberg A. M. Kaschak M. P. (2002). Grounding language in action. Psychon. Bull. Rev. 9, 558–565 12412897 10.3758/bf03196313

[B26] Glenberg A. M. Sato M. Cattaneo L. Riggio L. Palumbo D. Buccino G. (2008). Processing abstract language modulates motor system activity. Q. J. Exp. Psychol. 61, 905–919 10.1080/17470210701625550 18470821

[B27] Glover S. Dixon P. (2002). Semantics affect the planning but not control of grasping. Exp. Brain Res. 146, 383–387 10.1007/s00221-002-1222-6 12232695

[B28] Hasegawa M. Carpenter P. A. Just M. A. (2002). An fMRI study of bilingual sentence comprehension and workload. Neuroimage 15, 647–660 10.1006/nimg.2001.1001 11848708

[B29] Hasson U. Glucksberg S. (2006). Does negation entail affirmation? The case of negated metaphors. J. Pragmat. 38, 1015–1032

[B30] Hauk O. Davis M. H. Pulvermuller F. Marslen-Wilson W. D. (2006). The time course of visual word recognition as revealed by linear regression analysis of ERP data. Neuroimage 30, 1383–1400 10.1016/j.neuroimage.2005.11.048 16460964

[B31] Hauk O. Shtyrov Y. Pulvermüller F. (2008). The time course of action comprehension in the brain as revealed by cortical neurophysiology. J. Physiol. Paris 102, 50–58 10.1016/j.jphysparis.2008.03.013 18485679 PMC2441775

[B32] Horn L. R. (2001). A Natural History of Negation. Stanford: CSLI Publications

[B33] Kaup B. Lüdtke J. Zwaan R. A. (2006). Processing negated sentences with contradictory predicates: is a door that is not open mentally closed? J. Pragmat. 38, 1033–1050

[B34] Kaup B. Yaxley R. H. Madden C. J. Zwaan R. A. Lüdtke J. (2007). Experiential simulations of negated text information. Q. J. Exp. Psychol. 60, 976–990 10.1080/17470210600823512 17616914

[B35] Kemmerer D. Gonzalez-Castillo J. (2010). The two-level theory of verb meaning: an approach to integrating the semantics of action with the mirror neuron system. Brain Lang. 112, 54–76 10.1016/j.bandl.2008.09.010 18996582 PMC2859696

[B36] Kiefer M. Pulvermüller F. (2012). Conceptual representations in mind and brain: theoretical developments, current evidence and future directions. Cortex 48, 805–825 10.1016/j.cortex.2011.04.006 21621764

[B37] Kirk R. E. (1968). Experimental Design: Procedures for the Behavioral Sciences. Belmont, CA: Wadsworth

[B38] Kousta S. T. Vigliocco G. Vinson D. P. Andrews M. Del Campo E. (2011). The representation of abstract words: why emotion matters. J. Exp. Psychol. Gen. 140, 14–34 10.1037/a0021446 21171803

[B39] Kumar U. Padakannaya P. Mishra R. K. Khetrapal C. L. (2013). Distinctive neural signatures for negative sentences in Hindi: an fMRI study. Brain Imaging Behav. 7, 91–101 10.1007/s11682-012-9198-8 22869007

[B40] Lakoff G. Johnson M. (1980). Metaphors We Live By. Chicago, IL: University of Chicago Press

[B41] Lakoff G. Johnson M. (1999). Philosophy in the Flesh: The Embodied Mind and its Challenge to Western Thought. New York, NY: Basic Books

[B42] Liuzza M. T. Candidi M. Aglioti S. M. (2011). Do not resonate with actions: sentence polarity modulates cortico-spinal excitability during action-related sentence reading. PLoS ONE 6:e16855 10.1371/journal.pone.0016855 21347305 PMC3037953

[B43] Mahon B. Z. Caramazza A. (2008). A critical look at the embodied cognition hypothesis and a new proposal for grounding conceptual content. J. Physiol. 102, 59–70 10.1016/j.jphysparis.2008.03.004 18448316

[B44] Niedenthal P. M. Winkielman P. Mondillon L. Vermeulen N. (2009). Embodied emotion concepts. J. Pers. Soc. Psychol. 96, 1120–1136 10.1037/a0015574 19469591

[B45] Papeo L. Corradi-Dell'Acqua C. Rumiati R. I. (2011). “She” is not like “I”: the tie between language and action is in our imagination. J. Cogn. Neurosci. 23, 3939–3948 10.1162/jocn_a_00075 21671735

[B46] Penolazzi B. Hauk O. Pulvermüller F. (2007). Early semantic context integration and lexical access as revealed by event-related potentials. Biol. Psychol. 74, 374–388 10.1016/j.biopsycho.2006.09.008 17150298

[B47] Pulvermüller F. (1999). Words in the brain's language. Behav. Brain Sci. 22, 253–336 11301524

[B48] Pulvermüller F. (2004). Lexical access as a brain mechanism. Behav. Brain Sci. 27, 297–298 10.1017/S0140525X04260071 18241510

[B49] Pulvermüller F. (2005). Brain mechanisms linking language and action. Nat. Rev. Neurosci. 6, 576–582 10.1038/nrn1706 15959465

[B50] Pulvermüller F. Hauk O. Nikolin V. V. Ilmoniemi R. J. (2005a). Functional links between language and motor systems. Eur. J. Neurosci. 21, 793–797 10.1111/j.1460-9568.2005.03900.x 15733097

[B51] Pulvermüller F. Shtyrov Y. Ilmoniemi R. (2005b). Brain signature of meaning access in action word recognition. J. Cogn. Neurosci. 17, 884–892 10.1162/0898929054021111 15969907

[B52] Raposo A. Moss H. E. Stamatakis E. A. Tyler L. K. (2009). Modulation of motor and premotor cortices by actions, action words and action sentences. Neuropsychologia 47, 388–396 10.1016/j.neuropsychologia.2008.09.017 18930749

[B53] Ruschemeyer S. A. Brass M. Friederici A. D. (2007). Comprehending prehending: neuralcorrelates of processing verbs with motor stems. J. Cogn. Neurosci. 19, 855–865 10.1162/jocn.2007.19.5.855 17488209

[B54] Stins J. F. Beek P. J. (2013). Effects of language processing on spontaneous muscle activity. J. Neurolinguist. 26, 363–369 2615371

[B55] Tamietto M. Castelli L. Vighetti S. Perozzo P. Geminiani G. Weiskrantz L. (2009). Unseen facial and bodily expressions trigger fast emotional reactions. Proc. Natl. Acad. Sci. U.S.A. 106, 17661–17666 10.1073/pnas.0908994106 19805044 PMC2764895

[B56] Tassinary L. G. Cacioppo J. T. Vanman E. (2007). The skeletomotor system: surface electromyography, in The Handbook of Psychophysiology, 3rd Edn., eds Cacioppo J. T. Tassinary L. G. Bernston G. (New York, NY: Cambridge University Press), 267–299

[B57] Tettamanti M. Buccino G. Saccuman M. C. Gallese V. Danna M. Scifo P. (2005). Listening to action-related sentences activates fronto-parietal motor circuits. J. Cogn. Neurosci. 17, 273–281 10.1162/0898929053124965 15811239

[B58] Tettamanti M. Manenti R. Della Rosa P. A. Falini A. Perani D. Cappa S. F. (2008). Negation in the brain: modulating action representations. Neuroimage 43, 358–367 10.1016/j.neuroimage.2008.08.004 18771737

[B59] Tomasino B. Weiss P. H. Fink G. R. (2010). To move or not to move: imperatives modulate action-related verb processing in the motor system. Neuroscience 169, 246–258 10.1016/j.neuroscience.2010.04.039 20420884

[B60] Vigliocco G. Vinson D. P. Druks J. Barber H. Cappa S. F. (2011). Nouns and verbs in the brain: a review of behavioural, electrophysiological, neuropsychological and imaging studies. Neurosci. Biobehav. Rev. 35, 407–426 10.1016/j.neubiorev.2010.04.007 20451552

[B61] Winkielman P. Niedenthal P. M. Oberman L. (2008). The embodied emotional mind, in Embodied Grounding: Social, Cognitive, Affective, and Neuroscientific Approaches, eds Semin G. R. Smith E. R. (New York, NY: Cambridge University Press), 263–288

[B62] Zwaan R. A. Taylor L. J. Jr. (2006). Seeing, acting, understanding: motor resonance in language comprehension. J. Exp. Psychol. Gen. 135, 1–11 10.1037/0096-3445.135.1.1 16478313

